# Whole-Genome Sequence of a Novel Sequence Type 3136 Carbapenem-Resistant Klebsiella pneumoniae Strain Isolated from a Hospitalized Patient in Durban, South Africa

**DOI:** 10.1128/MRA.01300-18

**Published:** 2018-12-13

**Authors:** Yogandree Ramsamy, Koleka P. Mlisana, Mushal Allam, Arshad Ismail, Ravesh Singh, Daniel G. Amoako, Sabiha Y. Essack

**Affiliations:** a School of Laboratory Medicine and Medical Science, Department of Medical Microbiology, University of KwaZulu-Natal, Durban, South Africa; b National Health Laboratory Service, Sandringham, South Africa; c Sequencing Core Facility, National Institute for Communicable Diseases, National Health Laboratory Service, Sandringham, South Africa; d Antimicrobial Research Unit, College of Health Sciences, University of KwaZulu-Natal, Durban, South Africa; University of Maryland School of Medicine

## Abstract

Here, we describe the genome sequence of a novel sequence type 3136 (ST3136) Klebsiella pneumoniae strain isolated in South Africa. The 5,574,236-bp genome harbored 23 resistance determinants and 12 virulence factors that are of cardinal importance to infections. The genomics of Klebsiella pneumoniae offer valuable insights into its pathogenicity.

## Abstract (precis)

Here, we describe the genome sequence of a novel sequence type 3136 (ST3136) Klebsiella pneumoniae strain isolated in South Africa. The 5,574,236-bp genome harbored 23 resistance determinants and 12 virulence factors that are of cardinal importance to infections.

## ANNOUNCEMENT

Klebsiella pneumoniae is an encapsulated, nonmotile, Gram-negative pathogen of great medical significance that is implicated in a wide variety of infections in humans, including urinary tract infections, pneumonia, bacteremia, meningitis, and liver abscesses (1, 2). Carbapenem-resistant Enterobacteriaceae species, such as K. pneumoniae, are included in the World Health Organization global priority pathogen list, and carbapenems are usually the last-resort antibiotic by virtue of their broad spectrum of activity (3, 4). Here, we present the emergence of sequence type 3136 (ST3136), a novel carbapenem-resistant sequence type isolated from a rectal swab of an adult female patient in an intensive care unit of a public hospital in Durban, South Africa.

A chromogenic screening medium, Chromid Carba Smart (bioMérieux, France), was used for the isolation of the strain. Phenotypic microbial identification and antibiotic susceptibility testing were performed using the Vitek 2 system (bioMérieux, France). The Rapidec Carba NP (bioMérieux, France) test was used to detect carbapenem hydrolysis by carbapenemase-producing bacteria. The strain was grown on nutrient agar (Oxoid, England) and incubated overnight at 37°C prior to genomic DNA extraction. The QIAamp DNA minikit (Qiagen, Germany) was used to extract the total genomic DNA. A paired-end library (2 × 300 bp) was prepared using a Nextera XT DNA sample preparation kit, and whole-genome sequencing (WGS) was carried out on a MiSeq machine (Illumina, USA). The sequenced reads (3,325,060 reads) were quality trimmed using Sickle version 1.33 (https://github.com/najoshi/sickle) and *de novo* assembled using SPAdes version 3.11 (5). All resultant contiguous sequences were then submitted to GenBank, where gene annotation was implemented using the NCBI Prokaryotic Genome Annotation Pipeline (PGAP) (6). Furthermore, the assembled genome was submitted to the Klebsiella multilocus sequence type (MLST) database at Institut Pasteur (Paris, France) (http://bigsdb.pasteur.fr/klebsiella/klebsiella.html) to assign the new sequence type (7). The resistance genes and virulence factors were predicted using ResFinder (8) through the GoSeqIt tools Web platform (https://www.goseqit.com/web-services/) and the VirulenceFinder database (http://www.mgc.ac.cn/VFs/) (9), respectively.

The assembled genome yielded 180 contiguous sequences of longer than 200 bp, covering 5,574,236 bp, with a G+C content of 57.37%, an *N*_50_ value of 103,476 bp, and a longest contig size of 283,489 bp. The total number of 5,771 genes predicted by PGAP includes 5,427 protein-coding genes, 235 pseudogenes, and 109 RNA genes. The novel sequence type was defined as ST3136 by the Klebsiella MLST database (identifier 6431). Acquired antibiotic resistance genes to aminoglycosides [*aac(6')Ib-cr, aac(3)-IIa, aac(6')Ib-cr, aadA16, aph(3'')-Ib, aph(3')-Ia, aph(6)-Id*, and *rmtC*), β-lactams (*bla*_CTX-M-15_, *bla*_NDM-1_, *bla*_OXA-1_, *bla*_SHV-1_, and *bla*_TEM-1B_), fluoroquinolones [*aac(6')-Ib-cr, oqxA, oqxB*, and *qnrB6*], fosfomycin (*fosA*), macrolides [*mph(A)*], phenicol (*catA1* and *catB4*), rifampin (*ARR-3*), sulfonamides (*sul1* and *sul2*), and trimethoprim (*dfrA27*) were found, which confirmed the ability of WGS to make accurate resistome predictions. The VirulenceFinder database determined the following virulence factors: type I fimbriae (*fimG*) and type III fimbriae (*mrkA, mrkB, mrkC, mrkD*, and *mrkF*), lipoproteins (*pulG, pulO*, and *pulS*), and capsular polysaccharides (*wza, wzc*, and *wzi*), which contribute to the bacterium’s ability to adhere to, lyse, and invade host tissues, respectively (10). The valuable information offered by genomics provides a good step toward a better understanding of the pathogenicity of multidrug-resistant strains.

This study was approved by the Biomedical Research Ethics Committee (approval BE453/15) of the College of Health Sciences, University of KwaZulu-Natal (UKZN).

### Data availability.

This whole-genome shotgun project has been deposited in DDBJ/ENA/GenBank under the accession no. QMCA00000000. The version described in this paper is version QMCA01000000. The raw sequencing reads have been submitted to the SRA under the accession no. SRR8079358.
